# Parental knowledge, attitudes, and hesitancy toward human papillomavirus vaccination in Saudi Arabia: an online cross-sectional study

**DOI:** 10.3389/fmed.2026.1687347

**Published:** 2026-03-25

**Authors:** Mohammed Shaikhomer, Houriah Y. Nukaly, Razan A. Dalloul, Jumanah Y. Nassar, Amal M. Kayali, Shayma Al-Rubaki, Asim M. Alshanberi, Safaa M. Alsanosi, Ehab A. Abo-Ali

**Affiliations:** 1 Department of Internal Medicine, Faculty of Medicine, King Abdulaziz University, Jeddah, Saudi Arabia; 2 General Medicine Practice Program, Batterjee Medical College, Jeddah, Saudi Arabia; 3 Department of Pharmacology and Toxicology, Faculty of Medicine, Umm Al Qura University, Makkah, Saudi Arabia; 4 Public Health and Community Medicine Department, Faculty of Medicine, University of Tanta, Tanta, Egypt; 5 Department of Community Medicine, General Medicine Practice Program, Batterjee Medical College, Jeddah, Saudi Arabia

**Keywords:** cervical cancer, genital warts, human papilloma virus, immunization, parental attitudes, public health, reproductive health, vaccination

## Abstract

**Background:**

Cervical cancer incidence is rising in Saudi Arabia, leading to the integration of the human papillomavirus vaccine into the national immunization program. Parental awareness and acceptance play a critical role in the vaccine’s uptake. The current study aimed to study parental knowledge, attitudes and hesitancy toward human papillomavirus vaccine in Saudi Arabia.

**Methods:**

A cross-sectional study was conducted among 675 parents (using 95% CI and 5% margin of error) of daughters aged 11–15 years across various regions in Saudi Arabia. Data were collected using a self-administered questionnaire assessing sociodemographic factors, knowledge levels, and attitudes regarding the mandatory HPV vaccination.

**Results:**

Only 43.5% of parents were aware of the mandatory HPV vaccination program for adolescent girls, although only 11% had heard of HPV vaccination prior to the survey. Fathers were significantly less knowledgeable than mothers [adjusted odds ratio (AOR) = 0.29; 95% confidence interval (CI): 0.14–0.60, *p* = 0.001], indicating a notable gender disparity in health engagement. Parents in health-related occupations had a higher knowledge level than those in non-health-related jobs or who were unemployed (AOR = 2.15, 95% CI: 1.02–4.50, *p* = 0.043). Mothers who had never undergone a Pap smear were less likely to have knowledge than those who had (AOR = 0.33, 95% CI: 0.24–0.65, *p* < 0.001). Older parents showed less favorable attitudes than younger ones (AOR = 0.45, 95% CI: 0.22–0.91, *p* = 0.025), and parents living in urban areas had more positive attitudes compared to those in rural areas (AOR = 2.30, 95% CI: 1.12–4.74, *p* = 0.024). A negative family history of cervical cancer was also associated with a less positive attitude (AOR = 0.35, 95% CI: 0.23–0.56, *p* = 0.030). Key concerns contributing to hesitancy included potential side effects (53.4%) and vaccine safety (42.5%).

**Conclusion:**

Despite national policy support, parental awareness and knowledge of the HPV vaccination in Saudi Arabia remain limited. Strengthening the role of healthcare providers and addressing gender-based engagement disparities are essential components of targeted educational strategies aimed at improving vaccine uptake and reducing cervical cancer risk.

## 1 Introduction

Globally, cervical cancer (CC) is the fourth most common cancer among women, with an estimated 604,000 new cases and 342,000 deaths in 2020 (1, 2). Persistent infection with high-risk types of human papillomavirus (HPV), particularly HPV types 16 and 18, is responsible for approximately 70% of CC cases (3). HPV is a sexually transmitted infection, and among the more than 200 known HPV types, 13 are classified as high-risk for cancer development (4).

The ideal age to initiate vaccination is 11 or 12 years, although it can be administered as early as 9 years of age (5, 6). Focusing on this age group is crucial for effective prevention, as the vaccine should be given prior to potential exposure to HPV through any form of sexual activity (7).

In the United States, three prophylactic HPV vaccinations are licensed for use: Gardasil 9 (HPV9), Gardasil (HPV4), and Cervarix (HPV2) (5). All three vaccines protect against HPV types 16 and 18, which are strongly associated with cervical cancer. Additionally, the HPV9 and HPV4 vaccines target types 6 and 11, which are responsible for most cases of genital warts. Moreover, the HPV9 vaccine offers extended protection against five additional high-risk HPV types: 31, 33, 45, 52, and 58 (8).

In Saudi Arabia, CC ranks as the ninth most common cancer among women (3), with HPV types 16 and 18 being the most frequently detected high-risk strains. Saudi Arabia has a government-funded healthcare system that provides free and universal access to healthcare services for all citizens and registered residents. Preventive programs, such as the HPV vaccination, are fully subsidized and distributed through public healthcare facilities and school-based programs. Unfortunately, awareness and participation in screening and vaccination programs remain low (9, 10). Several studies have shown that Saudi women often lack knowledge about HPV and neglect preventive screening methods such as the Pap smear (11–13).

In response to global evidence supporting the effectiveness of HPV vaccination, countries like England and Denmark have reported significant reductions in cervical cancer incidence and HPV-related conditions after implementing national immunization programs (14, 15). Saudi Arabia has similarly taken steps to address this issue. The Saudi Food and Drug Authority (SFDA) approved the use of HPV vaccinations (Cervarix and Gardasil) in 2010 for females aged 11–26 years. In 2019, the Ministry of Health (MOH) officially included HPV vaccination in the Saudi National Immunization Schedule, making the vaccine freely available to the public. Saudi Arabia currently uses the Gardasil-9 HPV vaccination for girls aged 9–14 years as part of the national immunization program since April 2022. The Ministry of Health later extended coverage to females aged 15–26 years, providing one or two doses free of charge through public health centers and school-based programs in coordination with the Ministry of Education (16–18).

Despite these policy advancements, the prevalence of HPV vaccination in Saudi Arabia remains low. A recent study reported that only 4% of the population had received the HPV vaccination (18). Among female healthcare workers, uptake was slightly higher, at 20% (19), while a separate community-based survey reported 39.1% coverage (19). Based on the 4% coverage and a population of approximately 35 million, it is estimated that around 1.4 million individuals in Saudi Arabia have been vaccinated to date. This reflects a critical gap between policy and practice and underscores the need for greater parental awareness and public engagement.

Parental perception and acceptance are particularly crucial in a conservative society where parents make most healthcare decisions for their children. A previous national study conducted between 2019 and 2020 found that 70.6% of parents were unaware of the vaccine’s role in preventing HPV infection, and 89.5% of participants had not received the vaccine (19). It is important to note that this study took place during the early stages of HPV vaccination introduction in Saudi Arabia, which may explain the limited awareness and low uptake at the time. Nevertheless, the findings underscore the need for targeted public health education and engagement. Given the rising burden of HPV-related diseases and the low vaccine coverage, there is a pressing need to explore barriers to HPV vaccination uptake (20, 21).

Given the rising incidence of CC in Saudi Arabia, the strong association between HPV and cervical cancer, and the low vaccine coverage despite national policy endorsement, there is a clear need to explore barriers to vaccine acceptance. Understanding parental perceptions and attitudes toward mandatory HPV vaccination is essential for shaping effective health education and policy implementation. Thus, this study was conducted to assess parental perspectives on the mandatory HPV vaccination program for adolescent girls in Saudi Arabia. While previous studies have assessed awareness or acceptance of the HPV vaccination, this study uniquely analyzes the interplay of knowledge, attitudes, and hesitancy among Saudi parents, using a representative cross-sectional approach across all regions.

## 2 Materials and methods

### 2.1 Study design, setting, population, and sampling method

This cross-sectional study was conducted across various regions in Saudi Arabia, including urban and rural areas, encompassing the Central, Eastern, Northern, Southern, and Western provinces, to ensure a representative sample. The study participants included all parents of daughters aged between 11 and 15 years (as it matches Saudi MOH HPV vaccination target group) who agreed to participate in the study. Parents with daughters who were homeschooled online or unschooled were excluded.

Participants were informed about the study’s aims within the questionnaire and made aware that their involvement was voluntary and confidential. The minimum required sample size was calculated using the single-proportion formula with a 95% confidence interval and a 5% margin of error, assuming a 50% response proportion (due to lack of prior national data). This yielded 384 participants; the final sample was increased to 675 to improve precision and representativeness. Data collection occurred over 8 months (January to August 2023) via an online electronic questionnaire distributed using Google Form. Electronic informed consent was obtained via online form. Data were collected from participants residing in Jeddah, Riyadh, Makkah, Dammam, Abha, Tabuk, and other cities across all five regions. Distribution per region: Central (*n* = 130), Western (*n* = 185), Eastern (*n* = 110), Northern (*n* = 120), Southern (*n* = 130). Convenience sampling was employed through social media platforms and community outreach. While this approach allowed broad access, it is important to note that convenience sampling may introduce selection bias and limit the generalizability of findings.

### 2.2 Study tool

The questionnaire, adapted from Balogun and Grandahl’s tools, covered knowledge (10 items), attitudes (6 items), and perceptions (12 items) toward HPV vaccination, in addition to sociodemographic variables (22, 23), with modifications tailored to the Saudi context. The questionnaire was originally developed in English and translated into Arabic by the research team, all of whom are native Arabic speakers and proficient in English. A back-translation into English was then performed independently by a bilingual expert. Both versions were compared to resolve any discrepancies and ensure the accuracy and cultural relevance of the translated questionnaire.

To confirm content validity, a panel of experts in public health and epidemiology reviewed the questionnaire, providing feedback that was incorporated into the final version. The reliability of the questionnaire was tested on a pilot sample of 30 participants (excluded from the main study), yielding a satisfactory Cronbach’s alpha of 0.81 for knowledge and 0.84 for attitude.

The questionnaire comprised 37 items divided into three sections:

- The first section collected sociodemographic and health-related data about the parents and their daughters, including age, relation to the daughter, education, occupation, monthly income, residence, vaccination history, screening history, and awareness of the HPV vaccination.
- The second section assessed parental perceptions of HPV vaccination and hesitancy, awareness of HPV, cervical cancer, and vaccination, and attitudes toward the HPV vaccination. This section contained 18 items divided into three subsections.
- The third section evaluated parental knowledge about the mandatory HPV vaccination program for adolescent girls.

Knowledge was assessed via 10 true/false/do not know items on HPV risk factors, transmission, cervical cancer screening, vaccine dosage, and effectiveness. Correct answers were scored 1 point each. Attitudes were measured using 6 statements rated on a five-point Likert scale from 1 (strongly disagree) to 5 (strongly agree), resulting in scores ranging from 6 to 30.

Knowledge items (true/false/do not know):

1. HPV is a virus that can cause cervical cancer.
2. HPV is transmitted through sexual contact.
3. The HPV vaccination is part of the national immunization schedule in Saudi Arabia.
4. The vaccine is most effective if given before the onset of sexual activity.
5. The HPV vaccination can prevent genital warts.
6. Cervical cancer screening is necessary even after receiving the vaccine.
7. HPV infection can affect both males and females.
8. Only women need the HPV vaccination. (Reverse scored)
9. The HPV vaccination has been proven to be safe and effective.
10. A single dose of the HPV vaccination provides lifelong protection. (Reverse scored)

Attitude statements (Likert Scale):

1. I support including the HPV vaccination in the national program.
2. I would allow my daughter to receive the HPV vaccination.
3. The vaccine should be mandatory for school-aged girls.
4. I trust the safety of the HPV vaccination.
5. I believe the vaccine can prevent serious illness.
6. I am willing to recommend the HPV vaccination to others.

### 2.3 Variables

The independent variables were both the sociodemographic and health ones, while the dependent variables were the level of knowledge and attitudes. Knowledge was assessed using 10 true or false questions. Each correct answer was given a score of 1. A good knowledge level was defined as correctly answering at least 6 out of 10 items (≥60%), a threshold adopted from previous studies assessing HPV knowledge (22, 23).

Similarly, a positive attitude was defined as scoring 19 or more out of 30 on the attitude scale (~63%), consistent with benchmarks used in comparable vaccine acceptance studies (23). The total score ranged from 6 to 30. A score of 19 or more was categorized as a positive attitude, which is approximately 63% of the total score.

Perception was defined as beliefs or cognitive impressions regarding HPV vaccination safety, side effects, and social acceptability. It was measured through 12 statements using a 5-point Likert scale. Vaccine hesitancy refers to delay in acceptance or refusal of safe vaccines despite availability of vaccination services (WHO 2015). Vaccine hesitancy was assessed by the question: “In your opinion, which of the following could be the reasons for HPV vaccination hesitancy?”

### 2.4 Ethical considerations

The study was conducted in accordance with the Declaration of Helsinki and approved by the Institutional Review Board and Research Ethics Committee of Batterjee Medical College, Jeddah, Saudi Arabia (reference number: RES-2022-0060). Participants provided informed consent by agreeing to participate before completing the questionnaire. Confidentiality and the right to withdraw at any time were assured.

### 2.5 Statistical analysis

Data were analyzed using IBM SPSS version 26. Categorical variables were summarized using frequencies and percentages. Parental knowledge and attitude scores were dichotomized and analyzed as outcome variables.

Associations between independent variables and outcome variables were tested using chi-square tests. Variables with *p* < 0.2 in bivariate analyses were entered into the binary logistic regression model. A stepwise backward elimination method was used to retain significant predictors (*p* < 0.05). Binary logistic regression models were constructed to identify predictors of good knowledge and positive attitudes, reporting odds ratios with 95% confidence intervals. A *p* < 0.05 was considered statistically significant.

### 2.6 Study limitations

As a cross-sectional study, the findings reflect associations rather than causation and should be interpreted with caution. Convenience sampling may limit generalizability due to potential selection bias. The questionnaire, although adapted and validated, may still be influenced by social desirability or recall bias, especially in a culturally sensitive context like Saudi Arabia where parental decisions on vaccination are affected by sociocultural factors. Additionally, the percentages reported in Table 1 reflect multiple responses per participant, which should be considered when interpreting the data. Finally, the categorization of income levels may restrict direct comparisons with other studies due to variations in socioeconomic classification systems.

**Table 1:** Sociodemographic and health characteristics of participating parents of female adolescents in Saudi Arabia (*n* = 675).

Characteristic	*N* (%)
Relation to the daughter	
Mother	556 (82.4)
Father	119 (17.6)
Age group	
<40	372 (55.1)
40–49	227 (33.6)
≥50	76 (11.3)
Nationality	
Saudi	518 (76.7)
Non-Saudi	157 (23.3)
Residence	
Urban area	576 (85.3)
Rural area	99 (14.7)
Occupation	
Unemployed	377 (55.9)
Non-health-related job	222 (32.9)
Health-related job	76 (11.3)
Educational level	
High school and below	237 (35.1)
University/college and higher	438 (64.9)
Family income	
Enough and saving	132 (19.6)
Just enough	448 (66.4)
Not enough	95 (14.1)
Number of daughters	
1	194 (28.7)
2	230 (34.1)
3 or more	251 (37.2)
Number of daughters between 11 and 15	
1	474 (70.2)
2	138 (20.4)
3 or more	63 (9.3)
Offspring received routine vaccination	
Yes	558 (87.1)
No	87 (12.9)
Regular health check-up	
Yes	326 (48.3)
No	349 (51.7)
Performance of Pap smear by the mother	
Yes	207 (30.7)
No	468 (69.3)
Parental receipt of the HPV vaccine	
Yes	93 (13.8)
No	582 (86.2)
Family history of cervical cancer	
Yes	74 (11.0)
No	601 (89.0)

## 3 Results

The present study included 675 participants from all provinces of Saudi Arabia. The majority of participants (55.1%) were under the age of 40, followed by 33.6% in the age group of 40–50 and 11.3% aged 50 or above, as shown in Table 2. Most participants (76.7%) were Saudi, while 23.3% were non-Saudi. Most participants (85.3%) resided in urban areas, while 14.7% resided in rural areas. There was a statistically significant difference in attitude based on residence (*p* = 0.003), with a higher proportion of participants from urban areas having a positive attitude.

**Table 2:** Parental knowledge and attitude toward mandatory HPV vaccination in relation to sociodemographic and health characteristics in Saudi Arabia (n = 294).

Characteristic	Knowledge (*n* = 294)		*p*-Value	Attitude (*n* = 294)		*p*-Value
	Good *n* (%)	Poor *n* (%)		Positive *n* (%)	Negative *n* (%)	
Relation to the daughter						
Mother	124 (51.9)	115 (48.1)	0.005 ^*^	133 (55.7)	106 (44.4)	0.005 ^*^
Father	17 (30.9)	38 (69.1)		36 (65.5)	19 (34.6)	
Age group						
<40	79 (48.5)	84 (51.5)	0.192	92 (56.4)	71 (43.6)	0.075
40–49	43 (48.9)	45 (51.1)		44 (50.0)	44 (50.0)	
≥50	19 (44.2)	24 (55.8)		16 (37.2)	27 (62.8)	
Nationality						
Saudi	99 (51.0)	95 (49.0)	0.142	108 (55.7)	86 (44.3)	0.142
Non-Saudi	42 (42.0)	58 (58.0)		44 (44.0)	56 (56.0)	
Residence						
Urban area	118 (47.8)	129 (52.2)		137 (55.5)	110 (44.5)	0.003 ^*^
Rural area	23 (48.9)	24 (51.1)	0.884	15 (31.9)	32 (68.1)	
Occupation						
Unemployed	69 (43.1)	91 (56.9)	0.036 ^*^	85 (53.1)	75 (46.9)	0.813
Non-health-related job	40 (47.6)	44 (52.4)		43 (51.2)	41 (48.8)	
Health-related job	32 (64.0)	18 (36.0)		24 (48.0)	26 (52.0)	
Educational level						
High school and below	40 (44.4)	50 (55.6)	0.423	43 (47.8)	47 (52.2)	0.371
University/college and higher.	101 (49.5)	103 (50.5)		109 (53.4)	95 (46.6)	
Family income						
Enough and saving	42 (63.6)	24 (36.4)	0.004 ^*^	38 (57.6)	28 (42.4)	0.306
Just enough	88 (45.8)	104 (54.2)		99 (51.6)	93 (48.4)	
Not enough	11 (30.6)	25 (69.4)		15 (41.7)	21 (58.3)	
Number of daughters						
1	44 (49.4)	45 (50.6)	0.902	43 (48.3)	46 (51.7)	0.236
2	50 (46.3)	58 (53.7)		52 (48.2)	56 (51.9)	
3 or more	47 (48.5)	50 (51.6)		57 (58.8)	40 (41.2)	
Number of daughters between 11 and 15						
1	98 (50.0)	98 (50.0)	0.105	111 (56.6)	85 (43.4)	0.033 ^*^
2	27 (38.0)	44 (62.0)		32 (45.1)	39 (55.0)	
3 or more	16 (59.3)	11 (40.7)		9 (33.3)	18 (66.7)	
Offspring received routine vaccines						
Yes	128 (47.4)	142 (52.6)	0.525	140 (51.9)	130 (48.2)	0.862
No	13 (54.2)	11 (45.8)		12 (50.0)	12 (50.0)	
Regular health check-up						
Yes	50 (40.3)	74 (59.7)	0.025 ^*^	85 (50.0)	85 (50.0)	0.494
No	91 (53.5)	79 (46.5)		67 (54.0)	57 (46.0)	
Performance of Pap smear by the mother						
Yes	71 (59.2)	49 (40.8)	0.001 ^*^	67 (55.8)	53 (44.2)	0.239
No	70 (40.2)	104 (59.8)		85 (48.9)	89 (51.2)	
Parental receipt of the HPV vaccination						
Yes	35 (48.0)	38 (52.1)	0.998	33 (45.2)	40 (54.8)	0.200
No	106 (48.0)	115 (52.0)		119 (53.9)	102 (46.2)	
Family history of cervical cancer						
Yes	26 (47.3)	29 (52.7)	0.910	34 (61.8)	21 (38.2)	0.026 ^*^
No	124 (51.9)	115 (48.1)		108 (45.2)	131 (54.8)	

*Significant values.

Table 3 shows that most participants (55.9%) were unemployed, followed by 32.9% in non-health-related jobs and 11.3% in health-related jobs. The difference in knowledge based on occupation was statistically significant (*p* = 0.036), with a higher proportion of participants in health-related jobs having good knowledge. Most participants (64.9%) had a university/college education or higher, while 35.1% had a high school education or below. The majority of participants (82.4%) were mothers, while 17.6% were fathers. There was a statistically significant difference in knowledge (*p* = 0.005) and attitudes (*p* = 0.005) based on the relation to the daughter, with a higher proportion of mothers having positive knowledge and attitudes.

**Table 3:** Parental perception of mandatory adolescents’ HPV vaccination and related hesitancy in Saudi Arabia.

Perception of HPV vaccination and its related hesitancy		
Source of information	*n*	%
Media	163	24.1
Healthcare provider	79	11.7
Friends and family	70	10.4
My daughter’s school	61	9.0
Others	44	6.5
Reasons for vaccine hesitancy		
Expected side effects	157	23.3
Doubt about safety	125	18.5
Others	60	8.9
Child refusal for vaccination	52	7.7
Vaccine ineffectiveness	41	6.1
Expected costs	36	5.3
Refusal of all vaccines	31	4.6

Income distribution revealed that 66.4% reported just enough income, 19.6% had enough income with savings, and 14.1% reported insufficient income. Knowledge differed significantly by income category (*p* = 0.004), with those having enough income and savings scoring higher.

Approximately half of the respondents (51.7%) did not have regular health check-ups. Those with regular check-ups showed significantly more positive attitudes (*p* = 0.025). Pap smear performance was reported by 30.7%, with a significant association with knowledge levels (*p* = 0.001); women who had undergone Pap smears exhibited better knowledge. A large proportion (86.2%) had not received the HPV vaccination themselves. Most (89.0%) reported no family history of cervical cancer, though attitudes were significantly more negative among those without a family history (*p* = 0.026).

In this sample, 43.5% of parents had heard about the mandatory HPV vaccination, 47.96% demonstrated good knowledge, and 57.48% expressed a positive attitude.

Table 1 demonstrates the most common sources of information about the mandatory HPV vaccination and the reasons behind vaccine hesitancy among parents. The media (24.1%) and healthcare providers (11.7%) were the most frequently cited sources of information, followed by friends and family (10.4%), the daughter’s school (9.0%), and others (6.5%). Regarding hesitancy, the most common reasons reported were concerns about expected side effects (23.3%) and doubts about vaccine safety (18.5%). Other reasons included child refusal (7.7%), expected costs (5.3%), vaccine ineffectiveness (6.1%), and general refusal of all vaccines (4.6%).

**Figure 1:**
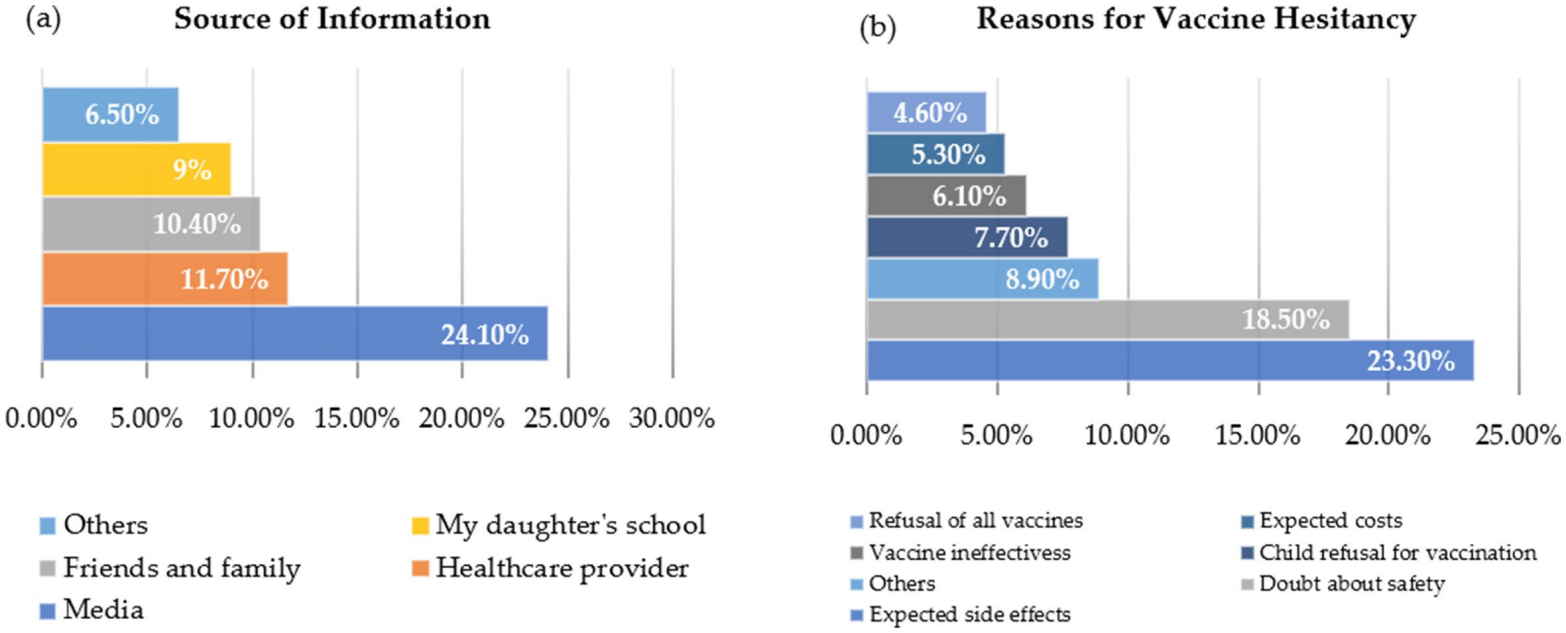
Representation of the results in Table 3. **(a)** The percentages of sources of information and **(b)** presenting the reasons for vaccine hesitancy.

Although participants were allowed to select multiple reasons for vaccine hesitancy, the total percentage is less than 100% because not all participants selected more than one reason. In terms of overall awareness, only 11.0% of parents had heard of HPV vaccination prior to completing the survey. However, 43.0% reported awareness of the mandatory HPV vaccination program for adolescent girls delivered through schools (see Figure 1).

The regression analysis results, presented in Table 4, revealed several significant factors influencing parental knowledge of the mandatory HPV vaccination in Saudi Arabia. Fathers were significantly less likely to possess good knowledge compared to mothers (AOR = 0.29, 95% CI: 0.14–0.60, *p* = 0.001). Parents working in health-related occupations were more likely to have good knowledge than those who were unemployed or employed in non-health-related fields (AOR = 2.15, 95% CI: 1.02–4.50, *p* = 0.043). In terms of income, parents with “just enough” income (AOR = 0.48, 95% CI: 0.26–0.91, *p* = 0.025) and those with “not enough” income (AOR = 0.24, 95% CI: 0.09–0.65, *p* = 0.005) were significantly less likely to have good knowledge compared to those with “enough income and savings.” Furthermore, mothers who had not undergone a Pap smear were less likely to be knowledgeable about the HPV vaccination than those who had (AOR = 0.33, 95% CI: 0.24–0.65, *p* < 0.001).

**Table 4:** Binary logistic regression analysis of good parental knowledge of the mandatory adolescents’ HPV vaccination in Saudi Arabia.

Variable	AOR	95% CI		*p*-value
Relation to the daughter				
Mother (ref)	1			
Father	0.29	0.14	0.60	0.001 *
Age group				
<40 (ref)	1			
40–49	1.04	0.57	1.89	0.899
≥50	0.73	0.35	1.54	0.408
Nationality				
Saudi (ref)	1			
Non-Saudi	0.77	0.42	1.39	0.383
Occupation				
Unemployed. (ref)	1			
Non-health-related job	1.27	0.69	2.33	0.445
Health-related job	2.15	1.02	4.50	0.043 *
Family income				
Enough and saving (ref)	1			
Just enough	0.48	0.26	0.91	0.025 *
Not enough	0.24	0.09	0.65	0.005 *
Number of daughters between 11 and 15 years				
1 (ref)	1			
2	0.62	0.34	1.16	0.135
3 or more	1.63	0.65	4.05	0.295
Regular health check-up				
Yes (ref)	1			
No	1.68	0.96	2.94	0.068
Performance of Pap smear by the mother				
Yes (ref)	1			
No	0.33	0.24	0.65	0.000 *

*Significant values.

Regarding attitudes toward HPV vaccination, as shown in Table 5, older parents (≥50 years) were less likely to express a positive attitude compared to those under 40 (AOR = 0.45, 95% CI: 0.22–0.91, *p* = 0.025). Urban residents were more likely to exhibit a positive attitude than those in rural areas (AOR = 2.30, 95% CI: 1.12–4.74, *p* = 0.024). Having multiple daughters aged 11–15 years was associated with a less favorable attitude: parents with two daughters (AOR = 0.55, 95% CI: 0.30–0.98, *p* = 0.042) or three or more (AOR = 0.36, 95% CI: 0.15–0.87, p = 0.024) were less likely to have a positive attitude compared to those with one daughter. Lastly, parents without a family history of cervical cancer were less likely to be supportive of the vaccine (AOR = 0.35, 95% CI: 0.23–0.56, *p* = 0.030).

**Table 5:** Binary logistic regression analysis of positive parental attitudes toward mandatory adolescent HPV vaccination in Saudi Arabia.

Variable	AOR	95% CI		*p*-Value
Relation to the daughter				
Mother (ref)	1			
Father	0.62	0.32	1.23	0.170
Age group				
<40 (ref)	1			
40–49	1.15	0.66	2.01	0.613
≥50	0.45	0.22	0.91	0.025 *
Nationality				
Saudi (ref)	1			
Non-Saudi	0.75	0.43	1.31	0.306
Residence area				
Rural (ref)	1			
Urban	2.30	1.12	4.74	0.024 *
Number of daughters between 11 and 15 years				
1 (ref)	1			
2	0.55	0.30	0.98	0.042 *
3 or more	0.36	0.15	0.87	0.024 *
Parental receipt of the HPV Vaccine				
Yes (ref)	1			
No	1.20	0.61	2.37	0.595
Family history of cervical cancer				
Yes (ref)	1			
No	0.35	0.23	0.56	0.030 *

*Significant value.

## 4 Discussion

This study focused on the awareness, knowledge, and attitudes of parents toward mandatory HPV vaccination in Saudi Arabia and provides essential insights into public health strategies for HPV vaccination and prevention programs. Our study found that a significant portion of Saudi and prevention programs. Our study found that a significant portion of Saudi Arabian parents, over half, were not familiar with the HPV vaccination. This lack of familiarity was also evident in a previous study in 2016 carried out on young women’s perceptions of HPV vaccination in Saudi Arabia (24–28).

The finding that 24.1% of parents cited media as their primary source of information about the HPV vaccination, compared to only 11.7% who reported healthcare providers, highlights a critical gap in clinical education. While the media can raise general awareness, it often lacks the accuracy and personalized guidance that healthcare professionals provide. This imbalance represents a missed opportunity for clinicians to engage directly with parents, address their concerns, and provide evidence-based information that could reduce vaccine hesitancy. To improve HPV vaccination uptake, educational interventions should prioritize strengthening the role of healthcare providers as trusted sources of information. This could include training healthcare professionals in effective communication strategies about HPV vaccination, integrating vaccine counseling into routine medical visits, and developing culturally appropriate educational materials for use in clinical settings (29, 30).

Healthcare providers need more support and training to confidently discuss the HPV vaccination, especially addressing safety concerns and cultural beliefs. By making vaccine talks a normal part of medical visits and creating materials that fit the local culture, clinicians can better guide parents. Involving fathers and working with community and religious groups can also help spread accurate information. These steps will help healthcare workers become stronger advocates and improve vaccine acceptance across Saudi Arabia.

The HPV vaccination was officially introduced into Saudi Arabia’s national immunization program as recently as 2019. Despite this, parental awareness and acceptance remain low. For example, a study in the Eastern Region of Saudi Arabia reported that while many parents were aware of the vaccine, hesitancy persisted due to concerns about vaccine safety and cultural beliefs linking the vaccine to early sexual activity (31, 32).

The current study revealed that vaccine hesitancy frequently arises from concerns about potential side effects and doubts about the vaccine’s safety. These findings are in agreement with those of other previous studies in different areas, as evidenced by a systematic literature review about HPV vaccine hesitancy in Europe. These studies revealed that the availability of information about the HPV vaccination and the potential side effects were the main reasons for vaccine hesitancy (33). In addition, vaccine hesitancy impacted the vaccination rate, as demonstrated in the study by Kiener et al. (34). This further emphasizes the need for healthcare professionals to engage more actively in educating the public about the benefits and importance of HPV vaccination for public health.

Parental attitudes toward HPV vaccination are strongly influenced by cultural and religious beliefs. In conservative societies like Saudi Arabia, concerns that HPV vaccination may encourage premarital sexual activity contribute to vaccine hesitancy. A systematic review highlighted that similar cultural concerns exist in multiple countries and represent a major barrier to acceptance (35, 36).

In our study, less than half of the participants who had heard about the HPV vaccination had comprehensive knowledge about HPV and its vaccine. This finding is aligned with recent studies in Saudi Arabia, which also showed the low level of knowledge and highlighted that the limited knowledge had contributed to parental hesitancy in vaccinating their daughters (32, 37). Factors influencing knowledge in our study included the participant’s relationship to the daughter, history of Pap smear test, occupation, and family income.

Our finding that fathers had 71% lower odds of being aware of the HPV vaccination compared to mothers reveals a significant gender disparity in healthcare engagement in Saudi Arabia. This gap highlights the need for targeted educational interventions specifically designed to reach and involve fathers in HPV vaccination efforts. Given that fathers often play a crucial role in family health decisions, increasing their awareness and knowledge is essential for improving vaccine uptake. Tailored strategies might include developing male-focused educational campaigns, involving fathers in healthcare visits, and leveraging community or workplace settings to disseminate information (38, 39). Addressing this gender gap can ensure that both parents are equally informed and supportive, thereby enhancing the effectiveness of public health initiatives related to HPV prevention.

Occupation played a role in knowledge levels; parents with health-related jobs were better informed about HPV. This could be explained by the likelihood that these parents acquired relevant knowledge in medical school or during their practice. This aligns with the findings of Rancic et al. (40), who showed higher HPV knowledge among those with medical education.

Parents with higher incomes were found to have better knowledge of HPV and its vaccine. This aligns with the findings of previous studies conducted in Ethiopia in 2021 and New Jersey in 2022, which similarly reported that socioeconomic status influences knowledge levels of HPV and its vaccination (41, 42). These results collectively suggest that higher income is often associated with greater access to education and health information. In contrast, a study conducted in China in 2018 reported no significant association between income and HPV knowledge levels (43). This difference may be partially explained by contextual factors such as the structure of the Chinese healthcare system, which may promote widespread access to public health education regardless of income level (44). However, this interpretation should be approached with caution, as health system impacts can vary and may not fully account for all observed differences.

It is worth noting that our study results show that the level of knowledge about HPV and its vaccine in Saudi Arabia appears to be low, regardless of residency area. On the other hand, other studies have found that parents residing in rural areas tend to have less knowledge about HPV and its vaccine compared to those living in urban areas (40, 45).

Despite the low parental knowledge levels, a relatively positive attitude toward vaccination was observed, with 57.48% of participants expressing positive attitudes. This finding falls between those reported by Hong Xie et al. (46) in China, where 87.6% were willing to vaccinate their children, and Seven et al. (47) in Turkey, where only 21.6% of mothers and 22.4% of fathers were willing (46, 47). Such variation in attitudes likely reflects cultural and educational differences.

The observed gaps in knowledge and the moderate level of positive attitudes require closer examination. For instance, only 47.9% of parents demonstrated good knowledge, a figure that aligns with prior national data but remains suboptimal given the availability of free vaccination. The finding that mothers had significantly greater knowledge and more positive attitudes than fathers reflects gendered differences in healthcare engagement, often driven by maternal involvement in pediatric care and reproductive health education. Additionally, attitude scores were higher among urban residents, consistent with previous studies in high-income countries where vaccine uptake correlates with accessibility, health literacy, and institutional trust (46, 47). Cultural factors such as conservatism and concern over sexual implications of the vaccine may also influence attitudes, especially in Middle Eastern contexts. These findings underscore the importance of culturally tailored education and increased clinical counseling to address vaccine hesitancy.

Factors associated with more positive attitudes toward vaccinating daughters included urban residency, younger age, a positive family history of cervical cancer, and having fewer daughters. However, these factors do not impact attitudes similarly in other countries, such as China, where Xie et al. (46) reported that age and residency had no significant effect on attitudes (46). Mothers generally showed more positive attitudes than fathers, a trend also observed in studies from Italy and China, suggesting cultural consistency across different societies (46, 48). Interestingly, parents with only one daughter showed more positive attitudes toward HPV vaccination than those with three or more daughters, possibly due to more focused parental care. Furthermore, a family history of cervical cancer correlated with more positive attitudes toward mandatory HPV vaccination.

While our study provides valuable insights into parental awareness, knowledge, and attitudes toward HPV vaccination in Saudi Arabia, certain limitations must be acknowledged. The study sample predominantly comprised urban residents and individuals with higher educational attainment, and the proportion of mothers was higher than the proportion of fathers. This demographic skew may limit the generalizability of our findings to the broader Saudi population, particularly those in rural areas, where healthcare access and educational resources may differ significantly. Future research should aim to include a more diverse and representative sample, encompassing various regions, educational backgrounds, and equal gender representation, to better understand and address the barriers to HPV vaccination across different segments of the Saudi population.

Saudi Arabia introduced the HPV vaccination into its national program in 2019 as part of Vision 2030. However, many parents remain unaware or unsure about the vaccine, with over half never having heard of it. This gap highlights a disconnect between policy and public awareness. Unlike in high-income countries with similar government-funded systems—such as Qatar or the UAE—Saudi Arabia still shows lower HPV vaccination coverage, suggesting that public health efforts need to better utilize healthcare infrastructure and culturally appropriate messaging.

Our findings also reveal disparities in knowledge, with urban residents, mothers, and parents from higher-income or medically educated backgrounds being more informed than others, particularly rural residents and fathers (40, 45). Tailored outreach is needed to reach these groups. Trusted community spaces, such as schools and religious centers, offer promising venues for vaccine education in Saudi Arabia’s conservative culture (46, 47). Overall, the program must focus not only on availability but also on accessibility, trust, and cultural sensitivity to increase vaccine uptake.

## 5 Conclusion

This study highlights the multifactorial nature of HPV vaccination hesitancy among Saudi parents, with knowledge, attitudes, and perceptions shaped by gender, income, health practices, and sociocultural influences. Although policy supports the HPV vaccination, uptake is hampered by insufficient parental knowledge and attitudinal concerns. Key findings revealed that only 43.5% of parents were aware of the vaccine mandate, 48% demonstrated good knowledge, and 57% showed a positive attitude. Fathers, rural residents, and lower-income families were less informed or supportive.

To improve HPV vaccination rates, educational campaigns must be tailored and multifaceted—leveraging accurate media messaging, school-based programs, and engagement with community leaders. Healthcare providers should be empowered and trained to address vaccine hesitancy through consistent, culturally sensitive counseling. While this study adds to the growing evidence supporting targeted interventions, it is limited by its cross-sectional design and potential selection and recall biases, as previously noted. Future studies should consider longitudinal designs and intervention-based approaches to better understand causal relationships and improve vaccination strategies.

## Data Availability

The original contributions presented in the study are included in the article/supplementary material, further inquiries can be directed to the corresponding author.

## References

[1] Arbyn M Weiderpass E Bruni L de Sanjosé S Saraiya M Ferlay J . Estimates of incidence and mortality of cervical cancer in 2018: a worldwide analysis. Lancet Glob Health. (2020) 8:e191–203. doi: 10.1016/S2214-109X(19)30482-6, 31812369 PMC7025157

[2] Sung H Ferlay J Siegel R Laversanne M Soerjomataram I Jemal A . GlobalCancerStatistics2020:GLOBOCANEstimatesofIncidenceandMortalityWorldwidefor36Cancersin185Countries. CA Cancer J Clin. (2021) 71:209–49. doi: 10.3322/caac.21660, 33538338

[3] Bruni L. Albero G. Serrano B. Mena M. Collado J. J. Gómez D. . Human Papillomavirus and Related Diseases in Saudi Arabia. Summary Report. (2023). Available online at: https://hpvcentre.net/statistics/reports/SAU.pdf) (Accessed March 6, 2025).

[4] Chrysostomou AC Stylianou DC Constantinidou A Kostrikis LG. Cervical cancer screening programs in Europe: the transition towards HPV vaccination and population-based HPV testing. Viruses. (2018) 10:729. doi: 10.3390/v10120729, 30572620 PMC6315375

[5] Meites E Szilagyi P Chesson H Unger E Romero J Markowitz L. Human papillomavirus vaccination for adults: updated recommendations of the advisory committee on immunization practices. MMWR Morb Mortal Wkly Rep. (2019) 68:698–702. doi: 10.15585/mmwr.mm6832a3, 31415491 PMC6818701

[6] Saslow D Andrews K Manassaram-Baptiste D Smith R Fontham E. Humanpapillomavirusvaccination2020guidelineupdate:AmericanCancerSocietyguidelineadaptation. CA Cancer J Clin. (2020) 70:274–80. doi: 10.3322/caac.21616, 32639044

[7] Vaccination Schedule for Saudi Arabia. Immunizationdata.who.int. (2022). Available online at: https://immunizationdata.who.int/pages/schedule-by-country/sau.html https://www.moh.gov.sa/en/HealthAwareness/EducationalContent/HealthTips/Documents/Immunization-Schedule.pdf (Accessed July 12, 2022).

[8] HPV Vaccination: What Everyone Should Know. Centers for Disease Control and Prevention;(2021). Available online at: https://www.cdc.gov/hpv/vaccines/index.html#:~:text=The%20vaccine%20is%20safe%20and,the%20cervix%20in%20young%20women (Accessed April 6, 2024).

[9] Obeid D Almatrrouk S Khayat H Al-Muammer T Tulbah A Albadawi I . Humanpapillomavirustype16and18viralloadsaspredictorsassociatedwithabnormalcervicalcytologyamongwomeninSaudiArabia. Heliyon. (2020) 6:e03473. doi: 10.1016/j.heliyon.2020.e03473, 32140590 PMC7047185

[10] Farahat F Faqih N Alharbi R Mudarris R Alshaikh S Al-Jifree H. Epidemiological characteristics of cervical cancer in a tertiary care hospital, western Saudi Arabia. Saudi Med J. (2021) 42:338–41. doi: 10.15537/smj.2021.42.3.20200603, 33632914 PMC7989268

[11] Almehmadi MM Salih MM Al-Hazmi AS. Awareness of human papillomavirus infection complications, cervical cancer, and vaccine among the saudi population: a cross-sectional survey. Saudi Med J. (2019) 40:555–9. doi: 10.15537/smj.2019.6.24208, 31219489 PMC6778758

[12] Akkour K Alghuson L Benabdelkamel H Alhalal H Alayed N AlQarni A . Cervical Cancer and human papillomavirus awareness among women in Saudi Arabia. Medicina. (2021) 57:1373. doi: 10.3390/medicina57121373, 34946318 PMC8707990

[13] Jradi H Bawazir A. Knowledge, attitudes, and practices among Saudi women regarding cervical cancer, human papillomavirus (HPV) and corresponding vaccine. Vaccine. (2019) 37:530–7. doi: 10.1016/j.vaccine.2018.11.065, 30503079

[14] Falcaro M Castañon A Ndlela B Checchi M Soldan K Lopez-Bernal J . The effects of the national HPV vaccination programme in England, UK, on cervical cancer and grade 3 cervical intraepithelial neoplasia incidence: a register-based observational study. Lancet. (2021) 398:2084–92. doi: 10.1016/S0140-6736(21)02178-4, 34741816

[15] Kjaer SK Dehlendorff C Belmonte F Baandrup L. Real-world effectiveness of human papillomavirus vaccination against cervical cancer. JNCI J Natl Cancer Inst. (2021) 113:1329–35. doi: 10.1093/jnci/djab080, 33876216 PMC8486335

[16] Farsi NJ Baharoon AH Jiffri AE Marzouki HZ Merdad MA Merdad LA. Human papillomavirus knowledge and vaccine acceptability among male medical students in Saudi Arabia. Hum Vaccin Immunother. (2021) 17:1968–74. doi: 10.1080/21645515.2020.1856597, 33522406 PMC8189128

[17] SabinVaccineInstitute. Advocating for Women’s Health in Saudi Arabia. (2023). Available online at: https://www.sabin.org/resources/advocating-for-womens-health-in-saudi-arabia (Accessed May 19, 2025).

[18] Obaiqy MA Mehdar SA Altayeb TI Saad TM Alqutub ST. Parental knowledge, views, and perceptions of human papilloma virus infection and vaccination-cross-sectional descriptive study. J Fam Med Prim Care. (2023) 12:556–60. doi: 10.4103/jfmpc.jfmpc_1673_22 PMC10131950 37122659

[19] Alqahtani AM Alshahrani MS Almajed E Salamah RA Alqntash N Biju AM . Human papillomavirus vaccination in Saudi Arabia: a narrative review of current evidence. J Pharm Bioallied Sci. (2024) 16:S4201–4. doi: 10.4103/jpbs.jpbs_798_24, 40061742 PMC11888674

[20] Alhusayn KO Alkhenizan A Abdulkarim A Sultana H Alsulaiman T Alendijani Y. Attitude and hesitancy of human papillomavirus vaccine among Saudi parents. J Fam Med Prim Care. (2022) 11:2909–16. doi: 10.4103/jfmpc.jfmpc_2377_21, 36119278 PMC9480641

[21] Suzuki Y Sukegawa A Ueda Y Sekine M Enomoto T Melamed A . The effect of a web-based cervical cancer survivor’s story on parents’ behavior and willingness to consider human papillomavirus vaccination for daughters: randomized controlled trial. JMIR Public Health Surveill. (2022) 8:e34715. doi: 10.2196/34715, 35421848 PMC9178460

[22] Balogun FM Omotade OO. Parental intention to vaccinate adolescents with HPV vaccine in selected communities in Ibadan, Southwest Nigeria: an application of Integrated Behavioral Model. Hum Vaccin Immunother. (2022) 18:2069959. doi: 10.1080/21645515.2022.2069959 35561294 PMC9359392

[23] Grandahl M Chun Paek S Grisurapong S Sherer P Tyden T Lundberg P. Parents’ knowledge, beliefs, and acceptance of the HPV vaccination in relation to their socio-demographics and religious beliefs: a cross-sectional study in Thailand. PLoS One. (2018) 13:e0193054. doi: 10.1371/journal.pone.0193054, 29447271 PMC5814087

[24] Hussain AN Alkhenizan A McWalter P Qazi N Alshmassi A Farooqi S . Attitudes and perceptions towards HPV vaccination among young women in Saudi Arabia. J Fam Community Med. (2016) 23:145–50. doi: 10.4103/2230-8229.189107, 27625580 PMC5009883

[25] Suarez-Lledo V Alvarez-Galvez J. Prevalence of health misinformation on social media: systematic review. J Med Internet Res. (2021) 23:e17187. doi: 10.2196/17187, 33470931 PMC7857950

[26] Kata A. A postmodern Pandora's box: anti-vaccination misinformation on the Internet. Vaccine. (2010) 28:1709–16. doi: 10.1016/j.vaccine.2009.12.022 20045099

[27] Wilson SL Wiysonge C. Social media and vaccine hesitancy. BMJ Glob Health. (2020) 5:e004206. doi: 10.1136/bmjgh-2020-004206, 33097547 PMC7590343

[28] Naoum P Athanasakis K Zavras D Kyriopoulos J Pavi E. Knowledge, perceptions and attitudes toward HPV vaccination: a survey on parents of girls aged 11–18 years old in Greece. Front Glob Women’s Health. (2022) 3:871090. doi: 10.3389/fgwh.2022.871090, 35783121 PMC9243232

[29] Opel DJ Mangione-Smith R Taylor JA Korfiatis C Wiese C Catz S . Development of a survey to identify vaccine-hesitant parents: the parent attitudes about childhood vaccines survey. Hum Vaccines. (2011) 7:419–25. doi: 10.4161/hv.7.4.14120, 21389777 PMC3360071

[30] Leask J Kinnersley P Jackson C Cheater F Bedford H Rowles G. Communicating with parents about vaccination: a framework for health professionals. BMC Pediatr. (2012) 12:154. doi: 10.1186/1471-2431-12-154, 22998654 PMC3480952

[31] Almutairi AB Alshehri M Almutairi R Aldosari M. Knowledge and attitude of parents toward human papillomavirus infection and HPV vaccination in Riyadh, Saudi Arabia. J Family Med Prim Care. (2025) 14:4871–4878. doi: 10.4103/jfmpc.jfmpc_252_25 41403521 PMC12704946

[32] Alkalash SH Alshamrani FA Alamer EH Alrabi GM Almazariqi FA Shaynawy HM . Parents’ knowledge of and attitude toward the human papilloma virus vaccine in the western region of Saudi Arabia. Cureus. (2022) 14:e32679. doi: 10.7759/cureus.32679 36660531 PMC9846376

[33] Karafillakis E Simas C Jarrett C Verger P Peretti-Watel P Dib F . HPV vaccination in a context of public mistrust and uncertainty: a systematic literature review of determinants of HPV vaccine hesitancy in Europe. Hum Vaccin Immunother. (2019) 15:1615–1627. doi: 10.1080/21645515.2018.1564436, 30633623 PMC6783136

[34] Kiener LM Schwendener CL Jafflin K Meier A Reber N Maurer SS . Vaccine hesitancy and HPV vaccine uptake among male and female youth in Switzerland: a cross-sectional study. BMJ Open. (2022) 12:e053754. doi: 10.1136/bmjopen-2021-053754, 35450894 PMC9024257

[35] Wilson R. HPV vaccine acceptance in West Africa: A systematic literature review. Vaccine. (2021) 38:5277–5284. doi: 10.1016/j.vaccine.2021.06.074 34366143

[36] Jiboc NM Paşca A Tăut D Băban AS. Factors influencing human papillomavirus vaccination uptake in European women and adolescents: a systematic review and meta-analysis. Psychooncology. (2024) 33:e6242. doi: 10.1002/pon.6242 37930064

[37] Alshehri MA Fahim WA Alsaigh RR Alshehri M Faheem WA. The association between parents’ knowledge about human papillomavirus and their intention to vaccinate their daughters: a cross-sectional study. Cureus. (2023) 15:e48600. doi: 10.7759/cureus.48600 38084180 PMC10710689

[38] Brewer NT Chapman GB Rothman AJ Leask J Kempe A. Increasing vaccination: putting psychological science into action. Psychol Sci Public Interest. (2017) 18:149–207. doi: 10.1177/1529100618760521, 29611455

[39] Glanz JM Wagner NM Narwaney KJ Shoup JA McClure DL McCormick EV . A mixed methods study of parental vaccine decision making and parent-provider trust. Acad Pediatr. (2013) 13:481–488. doi: 10.1016/j.acap.2013.05.030 24011751 PMC3767928

[40] Rancic NK Miljkovic PM Deljanin ZM Marinkov-Zivkovic EM Stamenkovic BN Bojanovic MR . Knowledge about HPV infection and the HPV vaccine among parents in southeastern Serbia. Medicina. (2022) 58:1697. doi: 10.3390/medicina58121697, 36556899 PMC9785943

[41] Dereje N Ashenafi A Abera A Melaku E Yirgashewa K Yitna M . Knowledge and acceptance of HPV vaccination and its associated factors among parents of daughters in Addis Ababa, Ethiopia: a community-based cross-sectional study. Infect Agents Cancer. (2021) 16:58. doi: 10.1186/s13027-021-00399-8, 34479576 PMC8418033

[42] Anuforo B McGee-Avila JK Toler L Xu B Kohler RE Manne S . Disparities in HPV vaccine knowledge and adolescent HPV vaccine uptake by parental nativity among diverse multiethnic parents in New Jersey. BMC Public Health. (2022) 22:195. doi: 10.1186/s12889-022-12573-7, 35093050 PMC8800253

[43] He J He L. Knowledge of HPV and acceptability of HPV vaccine among women in western China: a cross-sectional survey. BMC Womens Health. (2018) 18:130. doi: 10.1186/s12905-018-0619-8, 30053844 PMC6063014

[44] Wang L Wang Z Ma Q Fang G Yang J. The development and reform of public health in China from 1949 to 2019. Glob Health. (2019) 15:45. doi: 10.1186/s12992-019-0486-6, 31266514 PMC6604346

[45] Yu Y Xu M Sun J Li R Li M Wang J . Human papillomavirus infection and vaccination: awareness and knowledge of HPV and acceptability of HPV vaccine among mothers of teenaged daughters in Weihai, Shandong, China. PLoS One. (2016) 11:e0146741. doi: 10.1371/journal.pone.0146741, 26766565 PMC4713086

[46] Xie H Zhu HY Jiang NJ Yin YN. Awareness of HPV and HPV vaccines, acceptance to vaccination and its influence factors among parents of adolescents 9 to 18 years of age in China: a cross-sectional study. J Pediatr Nurs. (2023) 71:73–8. doi: 10.1016/j.pedn.2023.03.007, 37028228

[47] Seven M Güvenç G Şahin E Akyüz A. Attitudes to HPV vaccination among parents of children aged 10 to 13 years. J Pediatr Adolesc Gynecol. (2015) 28:382–6. doi: 10.1016/j.jpag.2014.11.005, 26209868

[48] Pelucchi C Esposito S Galeone C Semino M Sabatini C Picciolli I . Knowledge of human papillomavirus infection and its prevention among adolescents and parents in the greater Milan area, northern Italy. BMC Public Health. (2010) 10:378. doi: 10.1186/1471-2458-10-378, 20584324 PMC2901377

